# Optimization of Flocculation/Coagulation Conditions of Coal Preparation Plant Tailings Using Chitosan and FeCl_3_ Through Experimental Design

**DOI:** 10.3390/polym18060687

**Published:** 2026-03-12

**Authors:** Hasan Ali Taner, Augustino Henry Nyanswe

**Affiliations:** 1 Faculty of Engineering and Natural Sciences, Mining Engineering Department, Konya Technical University, Konya 42250, Türkiye; 2 Graduate Education Institute, Konya Technical University, Konya 42250, Türkiye; augustino.nyanswe@udom.ac.tz; 3 Department of Mining and Metallurgical Engineering, College of Earth Sciences and Engineering, The University of Dodoma, Dodoma 41107, Tanzania

**Keywords:** coal tailings, flocculation, chitosan, ferric chloride, water recovery, turbidity

## Abstract

Coal preparation plants generate large volumes of fine tailings containing negatively charged colloidal particles that remain stable in suspension and hinder efficient water recovery. In this study, the flocculation performance of coal tailings was statistically evaluated using inorganic and organic reagents, namely ferric chloride (FeCl_3_) and chitosan. The effects of chitosan dosage, FeCl_3_ dosage, pH, stirring speed, and pulp density on turbidity and water recovery were investigated through Response Surface Methodology (RSM). Zeta potential measurements revealed that the sample exhibited a negative surface charge over the entire pH range. In contrast, chitosan effectively shifted the surface charge toward positive values under acidic and near-neutral conditions, indicating charge neutralization and polymer bridging mechanisms. ANOVA results revealed that pH, chitosan dosage, and pulp density were the most significant parameters influencing turbidity and water recovery. Under optimized conditions, turbidity was reduced to 9.86 NTU with a water recovery of 76.92%. Using chitosan alone provided an effective and statistically validated strategy for dewatering samples by enhancing floc formation through combined charge neutralization and interparticle bridging mechanisms, resulting in minimal turbidity. Although chitosan alone was sufficient to achieve effective flocculation, its synergistic combination with FeCl_3_ resulted in the highest water recovery values under optimized conditions.

## 1. Introduction

Coal remains an important energy source worldwide due to its widespread availability and its role in energy security and electricity generation, particularly in developing and industrialized economies [1,2]. Türkiye’s strategic reliance on solid fuels continues to position the country as a major global stakeholder in coal production and reserves. In terms of international standing, Türkiye ranks 11th, with an estimated production of 1,671,453 TJ [3]. The latest exploration data from the General Directorate of Mineral Research and Exploration indicates that Türkiye’s total coal resources have approximately reached 21.8 billion tons, with lignite and asphaltite accounting for the vast majority at roughly 20.53 billion tons [4]. These reserves represent nearly 8–9% of the world’s total lignite, which positions the country among the top 5 global producers of lignite coal [5]. This is reflected in the national electricity operational data for 2025, which shows that preliminary production indicates approximately 80.5 million tons of lignite fuel for a fleet of 69 coal-fired power plants [6].

Low-rank coals are used when necessary to meet energy demand. Coal preparation processes, such as crushing, screening, and washing, are required to remove impurities and increase the calorific value of the coal; however, this ultimately leads to the generation of significant tailings. Furthermore, large amounts of water are used in coal preparation plants in a variety of these processes. One of the biggest challenges in these plants is the successful recovery and reuse of the plant water. For every ton of washed coal obtained, 3 m^3^ of slime water is produced [7]. Given that the world’s annual coal production exceeds billions of tons, it is evident that large volumes of coal wastewater containing significant amounts of ultrafine particles are generated [8]. Furthermore, these tailings have a high clay and ash content (25–75%). Due to their high content of fine particles (50–85%—45 µm), colloids remain stable in suspension without sedimentation [9,10].

The wastewater generated in coal preparation plants needs to be effectively dewatered. Water recovery is mainly achieved with the help of thickeners, reagents such as flocculants or coagulants that are used to accelerate the settling of solids. Coagulants are chemicals added to the suspension to provide destabilization, usually having a charge opposite to the surface charge of the fine-grained solid. Coagulants are widely used due to their low cost and easy availability. However, they have environmental and health-related disadvantages such as contamination of water with metals, low biodegradability, and the formation of large amounts of sludge [11]. Inorganic salts (such as aluminum sulfate, polyaluminum chloride (PAC), iron (III) chloride, iron (III) sulfate, and magnesium chloride) are the most commonly used coagulants for solid–liquid separation of coal wastewater [8].

Flocculants are used to destabilize dispersed particles, forming large and settleable flocs through aggregation [12]. The most commonly used flocculants are polymer-based substances such as polyacrylamide [12,13,14,15]. In recent years, carboxymethyl cellulose (CMC), guar gum, starch, and chitosan have been commonly used as natural flocculants [8,10,16,17,18,19]. On the other hand, organic flocculants such as chitosan are attracting attention due to their greater effectiveness and non-toxic properties compared to inorganic flocculants [20]. Chitosan is a biopolymer obtained by deacetylation of chitin [21]. Maria et al. (2020) [16] compared chitosan with Al_2_(SO_4_)_3_ and polyaluminum chloride in the treatment of coal wastewater. It was determined that chitosan showed superior performance by resulting in complete turbidity removal at a lower dose compared to these two, when all reagents were optimized in the neutral pH range. Mahto and Mishra (2021) [22] obtained flocculation efficiencies of 88.15% and 81.36% for kaolin and coal dust, respectively, using a guar gum-based flocculant they synthesized for the flocculation of kaolin and coal suspensions. Mehta (2022) [23] synthesized a novel carboxymethyl cellulose flocculant for the flocculation of fine coal suspensions and achieved a maximum flocculation efficiency of 83.6%. Although synthetic flocculants obtained by grafting are suitable for dewatering coal suspensions, the cost of synthetic grafting agents is still too high [8].

Chitosan is a biopolymer characterized by a molecular structure rich in amino (–NH_2_) and hydroxyl (–OH) active functional groups. These groups impart significant flocculation, chelation, and ion-exchange capacities, making chitosan an effective natural cationic flocculant. The flocculation performance of chitosan is mainly attributed to adsorption–bridging mechanisms, electrostatic charge neutralization, and specific interactions between its functional groups and suspended particles. Through these mechanisms, dispersed colloidal substances are aggregated into larger flocs, which subsequently facilitate sedimentation [8,24,25].

For the treatment of suspensions containing fine particles, the combined use of coagulants and flocculants is commonly adopted to enhance dewatering efficiency and promote the formation of larger and denser flocs [26,27]. Zhang et al. (2018) [28] demonstrated that integrating inorganic coagulants, such as PAC or AlCl_3_, with chitosan results in a pronounced synergistic effect. This synergy is primarily attributed to charge neutralization by the metal salts and interparticle bridging facilitated by both the inorganic coagulant and chitosan. Such combined mechanisms significantly improve floc formation, sedimentation behavior, turbidity removal, and reduction in natural organic matter. Moreover, the incorporation of chitosan has been reported to increase the effective molecular weight of the flocculant system, thereby strengthening bridging interactions [8].

In mineral suspensions, flocculation efficiency is governed by a combination of surface, chemical, physicochemical, and hydrodynamic parameters. The surface charge characteristics of mineral particles, typically expressed in terms of zeta potential, play a decisive role, as they determine interparticle electrostatic interactions and the adsorption behavior of flocculants. Since surface charge is strongly pH-dependent, pH control is critical, particularly in systems containing oxide and silicate minerals. Pulp density also influences collision frequency and suspension rheology, thereby affecting floc growth and structure. In addition, the molecular weight, charge density, and dosage of the flocculant are key operational variables; excessive dosage may lead to surface saturation and restabilization, whereas insufficient dosage results in weak and poorly settleable flocs. Hydrodynamic conditions, including stirring speed, determine floc formation and breakage behavior. Collectively, these parameters control the formation of dense, permeable, and shear-resistant flocs required for efficient sedimentation and dewatering in mineral processing operations.

Various experimental design methods are employed to optimize these parameters. Design of Experiments (DOE) is a highly efficient approach that allows a substantial amount of information to be obtained with a limited number of experiments while investigating the variations in parameters [29]. One of the most frequently used design methods is response surface methodology (RSM) [30]. RSM is a combination of statistical and mathematical methods that are useful for experimental design, modeling, analysis of the effects of variables, and the optimization of engineering problems [31]. Due to these advantages, it has received significant attention in dewatering studies [32].

The originality of this study lies in its comprehensive and statistically driven methodology for evaluating the flocculation performance of coal tailings with respect to both chemical and operational parameters. Dosage of iron chloride as an inorganic coagulant and chitosan as an organic biopolymer, pH, stirring speed, and pulp density were determined as independent variables. The study models not only the individual effects of these parameters but also their binary interactions through Response Surface Methodology (RSM). Moreover, the evaluation of flocculation performance encompassed in terms of turbidity and water recovery, alongside the optimization of process conditions. In this respect, this research enhances the understanding of the floc formation mechanism in the dewatering of coal tailings, providing a scientific basis for a more efficient and controlled dewatering strategy.

## 2. Materials and Methods

### 2.1. Materials

**Figure 1:**
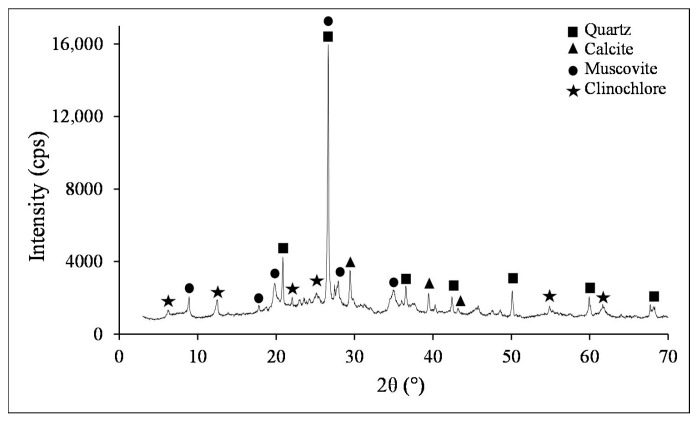
XRD pattern of the sample.

The tailings from the coal preparation plant were obtained from Ozsen Mining Company in Edirne/Türkiye. This material, collected as a suspension prior to being fed into the plant’s thickener, was transported to the laboratory and filtered. The filter cake was subsequently dried in an oven at 105 °C for 12 h, and sample preparation procedures were conducted. Representative samples were taken from this material to ensure homogeneity for use in characterization studies, and the remaining portion was packed for use in the experiments. In the characterization studies, the chemical composition was determined using X-ray fluorescence (XRF) analysis, and the mineralogical content was elucidated via X-ray diffraction (XRD) analysis. The chemical analysis results are presented in Table 1, indicating that the sample is a typical tailing with a relatively high SiO_2_ content. XRD pattern reveals that the sample predominantly consists of quartz, calcite, muscovite, and clinochlore phases (Figure 1). The presence of clay-related minerals such as muscovite and clinochlore is evident, signifying a substantial aluminosilicate component with layered structures. The major diffraction peak confirms the presence of quartz, consistent with the high SiO_2_ content determined by XRF analysis. The elevated Loss on Ignition (LOI) value is attributable to carbonate decomposition and residual carbonaceous matter. Overall, the mineral phases identified correspond well with the chemical data obtained from XRF analysis. Additionally, the structural features and morphology of flocs resulting from the sample and flocculation experiments were characterized using Fourier Transform Infrared Spectroscopy (FTIR) and Scanning Electron Microscopy (SEM).

**Figure 2:**
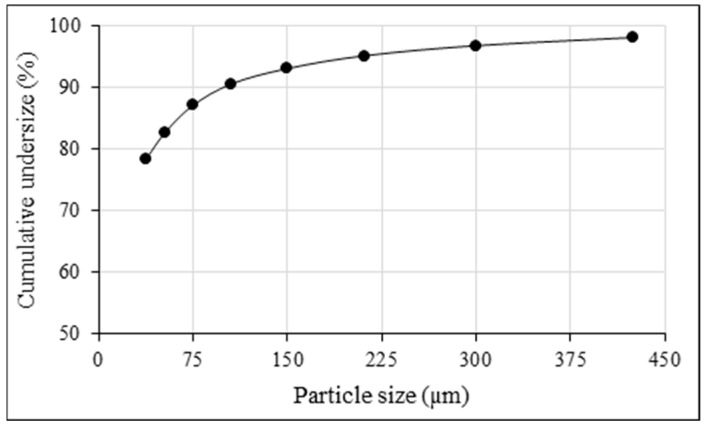
Particle size distribution of the sample.

The proximate analysis of the sample was conducted in accordance with ASTM standards, and the corresponding results are summarized in Table 2. The sample was subjected to wet sieving, and the d_90_ value was determined to be approximately 106 μm, as shown in Figure 2.

In the experiments, chitosan (Sigma-Aldrich, St. Louis, MO, USA)) was used as a flocculant and FeCl_3_ (Merck, Darmstadt, Germany) as a coagulant. The reagents were prepared daily at a concentration of 0.5% *w*/*v*, and stirred for 1 h using a magnetic stirrer at room temperature. Chitosan particles were dissolved in a 2% acetic acid solution. Analytical grade sodium hydroxide (NaOH) and hydrochloric acid (HCl) were utilized to adjust the pH of the suspension. All the experimental procedures were conducted at room temperature. The density of the sample was determined to be 2.2 g/cm^3^ using a pycnometer.

### 2.2. Methods

#### 2.2.1. Zeta Potential Analyses

Zeta potential (ζ-potential) measurements were conducted utilizing a ZetaPlus instrument (Brookhaven, Nashua, NH, USA) to acquire essential information concerning the adsorption selectivity of reactants on the mineral surface. The suspension was prepared to contain a concentration of 1 g/L sample and 10 mol/L sodium chloride (NaCl). NaOH and HCl were used to adjust the pH. The suspension was stirred using a magnetic stirrer for 15 min and subsequently allowed to settle for 5 min. A sample was taken from the upper phase of the suspension, following which ζ-potential measurements were performed. Each measurement was conducted three times on the same sample, and the mean values and standard deviations of the results were determined.

#### 2.2.2. Flocculation Experiments

The suspension, with varying pulp densities, was homogenized in a 0.5 L beaker for 5 min using a mechanical stirrer. Tap water was used in the flocculation experiments, and pH adjustment was carried out initially. Subsequently, reagents were added simultaneously in different dosages, and stirring was continued for 1 min. Afterward, the suspension was allowed to settle for 2.5 min, and the turbidity was measured by taking the supernatant from the top of the beaker. For this purpose, a Velp TB1 model turbidity meter based on the nephelometric turbidity unit (NTU) was used. Water recovery (%) was calculated by proportioning the mass of clear water obtained after flocculation to the initial total water mass.

#### 2.2.3. Multivariate Experimental Design

The experimental design (DOE) was performed using a statistical software program (Minitab v.17), and the Response Surface Method (RSM) was used for regression analysis of experimental data and for plotting the response surface. In this study, five independent variables were selected for the statistical experimental design based on preliminary experimental results. The independent variables and their respective levels, which influence the responses (turbidity and water recovery), are detailed in Table 3.

According to the experimental design, optimum conditions were estimated using a second-degree polynomial function that shows the correlation between the variables under investigation and the responses. The general form of this equation is as follows:

(1) $$Y\;=\;\beta_{0}+\sum_{i=1}^{k}\beta_{i}x_{i}+\sum_{i=1}^{k}\beta_{ii}{x_{i}}^{2}+\sum_{i<j}^{k}\beta_{ij}x_{i}x_{j}+\varepsilon$$

where *Y* is the predicted response variable, *β* is the coefficient, *x* is the independent variable, *k* is the number of variables, and *ε* is the error term. Analysis of variance (ANOVA) was used to estimate the statistical parameters. The fit of the experimental results to the model equation was expressed with the coefficient of determination R^2^, and the significance of the terms was estimated at a 95% confidence interval (*p* ≤ 0.05). The magnitude of the absolute values of the coefficients in front of the independent variables indicates the strength of their effects, while the “+” and “−” signs in front of the coefficients indicate whether the effect is positive or negative.

## 3. Results

### 3.1. Zeta Potential Studies

**Figure 3:**
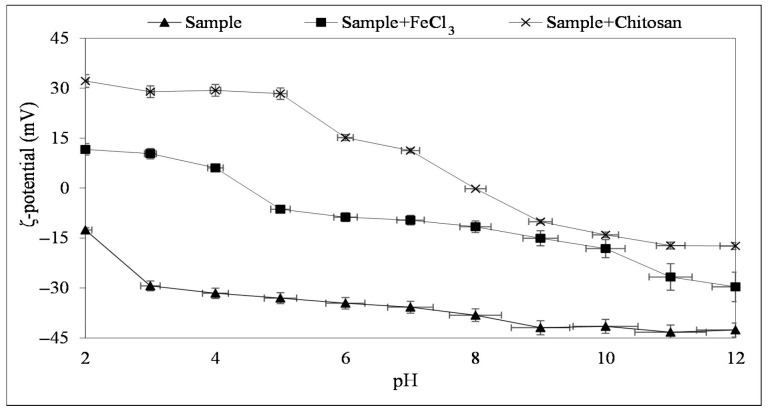
pH-dependent ζ-potential change in the sample, and the sample with FeCl_3_ and the sample with chitosan.

Zeta potential (ζ-potential) is regarded as one of the most common and effective electrokinetic parameters for understanding and interpreting the fundamental interaction mechanisms between mineral surfaces and flocculation reagents. To investigate the effect of the reagents used in flocculation experiments, the ζ-potentials of the sample, measured prior to and following the addition of reagents, were determined at varying pH levels (Figure 3). It was observed that the sample exhibited a negative surface charge throughout the pH range examined. As the pH increased, the surface charge became increasingly negative, indicating that the particles became electrostatically more stable, especially in an alkaline environment. The negative surface charge increased up to pH 9, reaching −42.62 mV at pH 12. Similar results were obtained by Kumar et al. (2014) [33] and Khatibi (2016) [12]. This phenomenon can be attributed to the presence of alumina and silicate-based particles, which can lead to lower negative ζ-potential values. The addition of FeCl_3_ shifted the surface charge to positive under low pH conditions; however, the ζ-potential rapidly decreased with increasing pH, reaching the isoelectric point around pH 4–5, and exhibiting negative values at higher pH values. This behavior suggests that Fe^3+^ ions provide effective charge neutralization at low pH, but their effectiveness decreases with increasing pH due to the hydroxyl complexes formed as a result of Fe^3+^ hydrolysis. When chitosan was incorporated, higher positive ζ-potential values were obtained at low pH, which decreased with rising pH, approaching the isoelectric point at approximately pH 8. This indicates that the protonated amino groups of chitosan effectively alter the surface charge in acidic environments, and as pH rises, the positive charge density decreases due to the deprotonation effect. Overall, within the same pH range, the ζ-potential values of the chitosan-added sample are more affected by increasing pH compared to the FeCl_3_-added sample. It appears that chitosan can effectively modify the surface charge of the sample and facilitate flocculation via both charge neutralization and polymer bridging mechanisms.

When the ζ-potential is zero, also known as the isoelectric point, the repulsive forces are weak, and the particles tend to gather and settle together. Likely charged particles repel each other, while oppositely charged particles attract one another [34]. Since the sample behaves differently at different pH values and in the presence of other ions, it can disperse or flocculate depending on the pulp conditions. Chitosan induces to flocculation by neutralizing the surface charges of the sample and forming a physical bridging.

### 3.2. Flocculation Studies

The conditions and results of the flocculation experiment were conducted in accordance with the experimental design matrix, which comprised 32 experiments as detailed in Table 4. Within the design matrix, variable levels were encoded as −1, 0, and +1, representing low, medium, and high values, respectively. Statistically significant differences were observed in the turbidity and water recovery values derived from the experimental results. Turbidity ranged from 15.25 NTU to 6485 NTU, while water recovery varied from 1.72% and 81.43%. The lowest turbidity value of 15.25 NTU was achieved at a stirring speed of 750 rpm, a pulp density of 5%, a pH of 5, with dosages of 200 g/t chitosan and 500 g/t FeCl_3_. Conversely, the highest water recovery of 81.43% was recorded at a stirring speed of 500 rpm, a pulp density of 5%, a pH of 8, with dosages of 100 g/t chitosan and 250 g/t FeCl_3_.

The experimental data were analyzed, and the models describing the turbidity and water recovery were obtained and are shown as Equations (2) and (3), respectively:

Turbidity (NTU) = 462.495 − 517.867A + 38.258B + 1739.5C − 81.053D + 1114.93E + 347.784A^2^ − 183.741B^2^ + 2028.06C^2^ − 243.991D^2^ − 214.866E^2^ − 115.835AB + 21.478AC + 45.347AD + 196.472AE − 124.165BC + 220.778BD + 36.278BE − 91.597CD + 1167.4CE + 83.585DE, R^2^ = 97.31% (2)

Water recovery (%) = 41.143 + 11.332A + 2.221B − 13.006C − 2.557D − 21.52E − 17.768A^2^ + 7.777B^2^ − 16.931C^2^ + 5.683D^2^ + 9.5E^2^ + 1.485AB + 4.694AC − 1.171AD − 6.632AE − 1.938BC − 8.553BD + 0.506BE + 1.946CD + 4.454CE − 1.03DE, R^2^ = 96.15% (3)

In the data analysis process, model validation is considered a critical component, as an inadequate model may result in misleading findings. The coefficient of determination (R^2^) demonstrates that the predictive models account for over 95% of the variance in the data. A higher coefficient of determination correlates with increased model reliability. The models indicate that increasing pH, FeCl_3_ dosage, and pulp density correlate with increased turbidity; conversely, increasing chitosan dosage and stirring speed correlate with decreased turbidity. In terms of water recovery, the models show that increasing chitosan and FeCl_3_ dosages enhances water recovery, whereas increasing pH, stirring speed, and pulp density diminishes water recovery.

Analysis of variance (ANOVA) was conducted to assess the impact of each variable on turbidity and water recovery, and the results are shown in Table 5. The ANOVA results indicate that turbidity is primarily influenced by pH (C), as evidenced by its notably high F-value. This underscores the essential role of pH in controlling surface charge characteristics of particles and the degree of protonation of chitosan. Given that chitosan is a cationic biopolymer, its charge density amplifies under acidic conditions, thereby increasing electrostatic attraction and charge neutralization. Chitosan dosage (A) and pulp density (E) were also significant factors for turbidity, suggesting that sufficient polymer concentration and collision frequency between particles are critical for effective bridging and aggregate formation. The statistical significance of the C^2^ term suggests a non-linear relationship, implying that there exists an optimal pH range in which turbidity decreases. Furthermore, the interaction between pH and pulp density (CE) was found to be significant. An increase in turbidity occurs due to the decreased likelihood of particle-polymer collisions. Conversely, FeCl_3_ dosage (B) and stirring speed (D) did not exhibit statistically significant effects on turbidity.

In consistent with the observed turbidity, pulp density (E), pH (C), and chitosan dosage (A) were found to be statistically significant factors affecting water recovery, displaying both linear and quadratic effects. This indicates that optimal polymer dosage and charge conditions are essential not only for particle aggregation but also for the formation of compact flocs. The significance of the quadratic terms A^2^ and C^2^ suggests that excessive chitosan dosing may result in overdosing effects, such as steric stabilization or the development of loose floc structures, which can adversely influence dewatering performance. The interaction terms signify that the impact of one operational parameter is dependent on the level of another. Water recovery is notably influenced by significant interaction factors (AC, AE, and BD). Specifically, the AC interaction demonstrates the pH-dependent nature of chitosan regarding charge neutralization and polymer bridging. Although FeCl_3_ dosage (B) did not exhibit a pronounced individual effect on turbidity, its interaction with stirring speed (BD) plays an important role in water recovery. It is postulated that the amorphous iron hydroxide compound formed through the hydrolysis of iron ions surrounds the particles, thereby maintaining them in suspension under suitable hydrodynamic conditions.

The stirring speed ensures homogeneous distribution of flocculants. Although the data in Table 5 indicate that stirring speed is an insignificant factor, it is observed that high-speed stirring diminishes turbidity but concurrently decreases water recovery, as described by Equations (2) and (3). While stirring speed is an insignificant factor compared to chitosan dosage, pH and pulp density, it is essential to identify the optimal stirring speed to prevent floc breakage and to ensure effective flocculation [8,35].

**Figure 4:**
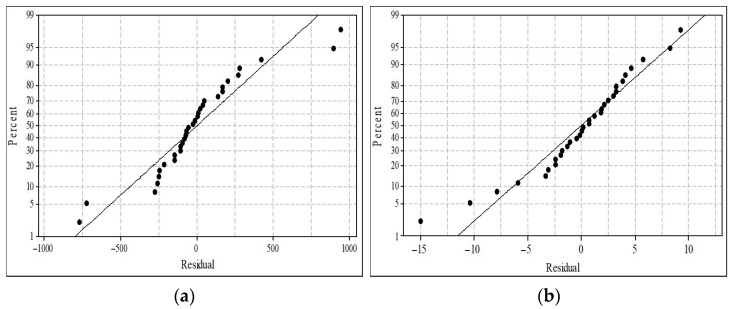
Normal probability plots of turbidity (**a**) and water recovery (**b**).

Normal probability plots, drawn to check the reliability of the statistical analysis, are given in Figure 4. The proximity of the experimental points to the reference line indicates adherence to a normal distribution and conformity with the model. This implies that the experiments are reliable. However, it is observed that a few points, particularly at the extremities, deviate from the linear trend. Since these deviations do not introduce systematic skewness, they are not severe enough to significantly undermine the model assumptions.

**Figure 5:**
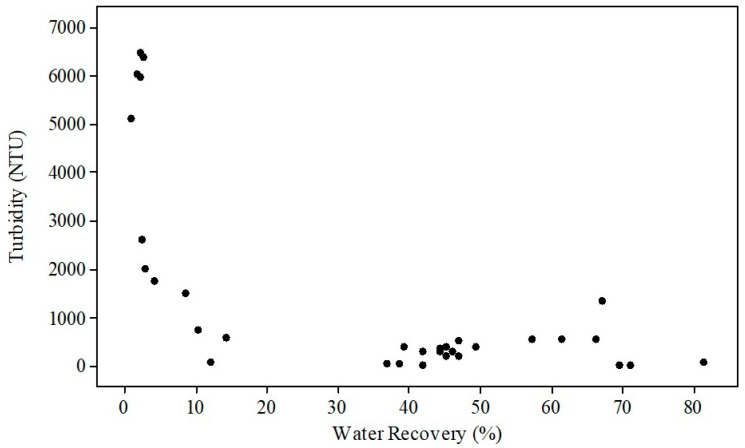
The relationship between turbidity and water recovery.

Figure 5 illustrates the relationship between turbidity and water recovery data obtained from flocculation experiments. An analysis of Figure 5 demonstrates a significant inverse correlation between these two responses. While turbidity is quite high (5000–6500 NTU) at low water recovery values (0–15%), it is observed that turbidity diminishes as water recovery increases. Specifically, for water recoveries of 40% and higher, turbidity values tend to cluster within a narrow range.

**Figure 6:**
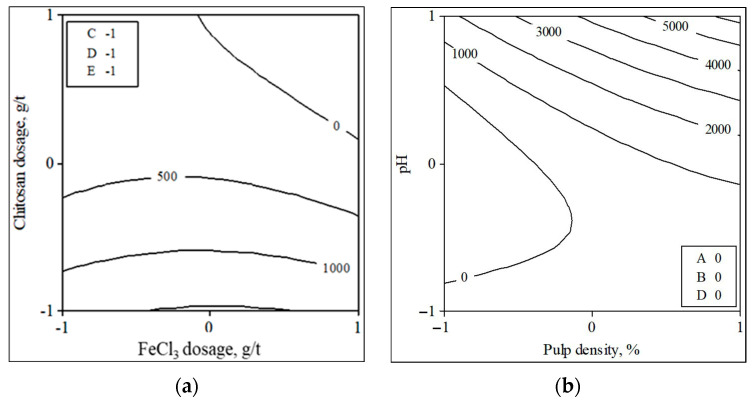
Contour plot of turbidity depending on chitosan–FeCl_3_ dosage (**a**) and pH–pulp density (**b**).

**Figure 7:**
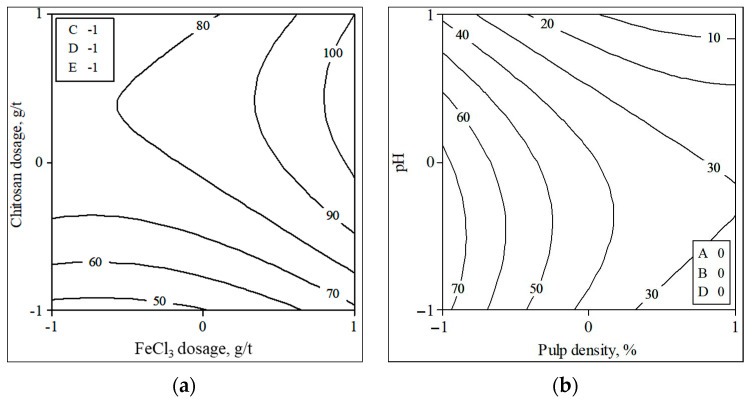
Contour plot of water recovery depending on chitosan–FeCl_3_ dosage (**a**) and pH–pulp density (**b**).

The contour plots illustrating the effects of variables on turbidity and water recovery are shown in Figure 6 and Figure 7. When the effect of the interaction between chitosan and FeCl_3_ concerning turbidity is examined, it is observed that the FeCl_3_ dosage exhibits a negligible effect, whereas turbidity diminishes with an increase in chitosan dosage. At the optimal polymer dosage, polymer chains adsorb onto multiple particle surfaces, thereby facilitating aggregation through effective inter-particle bridging. When the dosage falls below this optimal level, insufficient bridging sites result in incomplete floc formation and residual suspended particles. Conversely, an excessive addition of polymer results in surface saturation and steric stabilization, which inhibit effective bridging and decrease flocculation efficiency [12,36,37]. Therefore, it is necessary to use reagents at the optimal dosage where the surfaces of the particles are coated with polymer chains and the suspension becomes unstable. High pH and high pulp density increase turbidity. This can be attributed to the decreased solubility of chitosan at high pH values [38,39]. In experiments conducted at pH 11, turbidity reached up to 6000 NTU. This was due to the high concentration of hydroxyl ions in the suspension. As a result, the particles tend to repel each other, preventing aggregation [11,40,41]. Water recovery generally improves as the dosage of both reagents increases, but decreases with higher pH and pulp density. FeCl_3_ is economically advantageous due to its low unit cost, large-scale production and widespread industrial availability, whereas chitosan has a relatively higher chemical cost because of its biopolymeric origin and processing requirements. Nevertheless, the combined FeCl_3_–chitosan system may offer indirect economic and environmental benefits by reducing residual turbidity, enhancing water recovery, and partially replacing inorganic salts with a biodegradable polymer.

**Figure 8:**
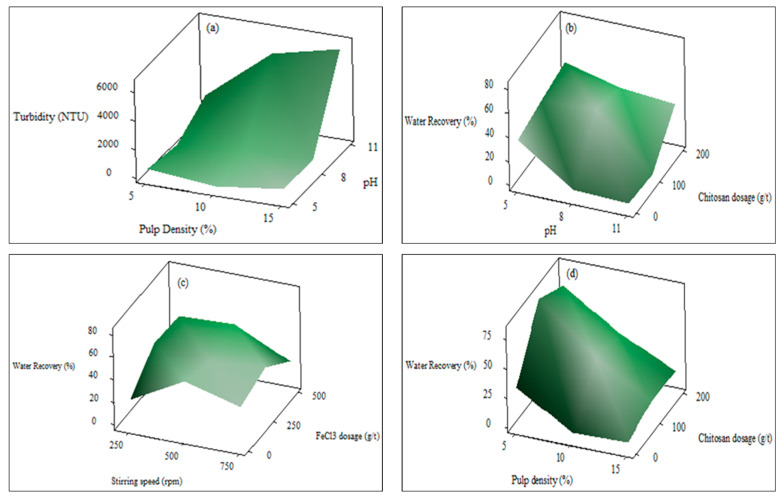
Response surface plots showing the effect of pulp density–pH on turbidity (**a**), the effects of pH–chitosan dosage (**b**), stirring speed–FeCl_3_ dosage (**c**), and pulp density–chitosan dosage (**d**) on water recovery.

Three-dimensional response surface plots of the effects of experimental factors on turbidity and water recovery are shown in Figure 8. These plots are used to examine the effect of either of the two variables on the response, while other variables are held at a moderate constant level. While the pulp density–pH interaction has a significant effect on turbidity, the interactions of chitosan dosage–pH, pH–pulp density, and FeCl_3_ dosage–stirring speed have significant effects on water recovery. Turbidity increased significantly with increasing pH and pulp density (Figure 8a). The lowest turbidity was observed at pH 5 and a pulp density of 5%. Higher solid content led to more suspended particles and increased collision frequency, resulting in a turbid suspension at steady state. Figure 8b shows that the water recovery exhibits nonlinear behavior depending on chitosan dosage and pH parameters. Water recovery remains low at low chitosan dosage and high pH, while it increases within a specific pH range with increasing dosage. The higher water recovery at moderate pH values suggests that the protonation degree of chitosan, and consequently its charge density, is more favorable in this range. While effective polymer bridging occurs under these conditions, it can be said that the floc structure loosens and the water-retention capacity increases due to surface saturation and potential steric stabilization effects in cases of overdose. This indicates that the optimum dosage of the polymer in suspension and pH adjustment are necessary. Solution pH controls the surface charge of colloidal particles and the ionization degree of the polymer. By altering polymer charge density and chain conformation, pH directly affects the optimum dosage and overall flocculation recovery [12,42].

When Figure 8c is examined, it is seen that the water recovery reaches its maximum at moderate stirring speeds and within a specific range of FeCl_3_ dosage. At low stirring speeds, floc formation may be limited due to the non-homogeneous distribution of the coagulant, while at excessively high speeds, the flocs formed may break, and their structural integrity may be compromised. Similarly, low FeCl_3_ dosage leads to stability, while excessive dosage can increase water retention due to excess metal hydroxide and reduced precipitation. Metal cations, such as Fe, can reduce the negative surface charge of the particles [12]. Positively charged FeCl_3_ is expected to adsorb to the surface of negatively charged particles, possibly via electrostatic attraction, leading to charge neutralization. Inorganic flocculants such as iron chloride form cationic metal hydroxides in water. These metal hydroxides support flocculation through mechanisms such as surface charge neutralization and sweep flocculation [20,43]. Figure 8d shows that water recovery reaches high values at low pulp densities and moderate chitosan dosages. The tendency for water recovery to decrease as pulp density increases can be attributed to less floc formation at high solid ratios and the high water-holding capacity of the flocs. At the same time, water recovery is low at low polymer dosages due to insufficient bridging.

Chitosan functions as a coagulant–flocculant through charge neutralization and polymer bridging mechanisms [16,21,44,45,46]. As a polycationic biopolymer, its amine groups become protonated under acidic conditions, facilitating the destabilization of negatively charged colloidal particles. Its long-chain structure further enhances inter-particle bridging, resulting in floc formation and subsequent sedimentation [16]. A study investigating the flocculation behavior of coal wastewater [8] concluded that the addition of AlCl_3_ and chitosan improved dewatering by augmenting electrical neutralization and bridging capabilities. When used at appropriate dosages, these reagents accelerate sedimentation by forming a compact floc structure.

### 3.3. Response Optimization

Turbidity and water recovery represent two distinct responses; therefore, optimizations have been conducted under different conditions. Table 6 exhibits the optimized flocculation parameters alongside the corresponding responses obtained through the experimental design. To diminish turbidity, the chitosan dosage should approach a high dosage, the stirring speed should be elevated, the pH should be maintained near a medium level, and both the FeCl_3_ dosage and pulp density should be kept at a low level. Conversely, to enhance water recovery, the chitosan dosage should be set at approximately 0.515, the FeCl_3_ dosage should be high, the pH should be at approximately −0.556, and both the stirring speed and pulp density should be maintained at low levels. Experiments were conducted under these conditions, yielding a turbidity value of 9.86 NTU and a water recovery of 76.92%. Although a turbidity of 15.25 NTU was achieved with the chitosan and FeCl_3_ combination due to charge neutralization and bridging synergies, this was a result of high total reagent consumption and the complexity of the reagent handling process. The turbidity of 9.86 NTU obtained under optimized conditions suggests that the use of chitosan in coal tailings dewatering, along with other suitable conditions, has strong potential for industrial applications. Flocculation experiments were performed at pH 7.55, determined as the optimal condition, rather than in alkaline or acidic environments. The negatively charged surfaces of quartz and phyllosilicates enhance electrostatic repulsion at neutral to alkaline pH, requiring effective charge neutralization. FeCl_3_ hydrolysis products provide multivalent cations that compress the electrical double layer, thereby promoting destabilization. Protonated amine groups (–NH_3_^+^) of chitosan interact strongly with negatively charged silicate surfaces. Since the mineralogical composition directly supports the proposed dual charge neutralization and polymer bridging mechanism, the best performance was achieved at the optimal pH value. Compared with results obtained with chitosan alone, the dual system showed higher predicted and experimentally validated water recovery values in the RSM optimization analysis.

**Figure 9:**
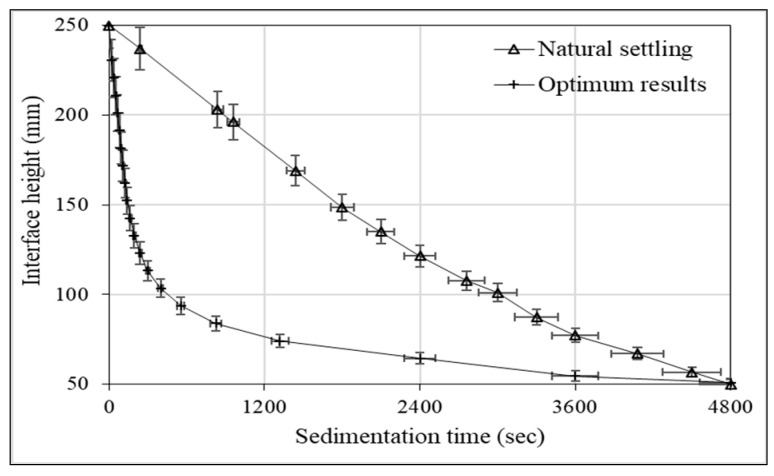
Time-dependent variation in interface height for natural and optimum settling conditions.

Figure 9 shows the variation in the interfacial height with sedimentation time before the addition of any reagent (natural settling) and under the optimum conditions determined for turbidity. In both cases, the interfacial height decreases with time. It is observed that the interfacial height decreases much more rapidly with chitosan under optimal conditions. A significant decrease was observed, particularly in the initial period, indicating that the settling rate increased significantly compared to natural settling due to floc formation. The settling rate of flocs is a critical parameter in the flocculation process and can directly affect process cost and efficiency [47,48]. This study demonstrates that chitosan performs the necessary functions for dewatering coal tailings in suspension.

Optimized reagent dosages and other parameters provide a preliminary operating range, while industrial thickeners often require pilot-scale validation. This study clearly indicates that pilot-scale validation under continuous flow conditions is a logical next step towards full-scale industrial application. What changes with scale is hydrodynamic behavior and operating parameters (such as feed flow rate, feed point design, stirring, sediment depth, rake speed, torque control, and underflow density), not the fundamental destabilization chemistry.

This study is expected to guide the literature by encouraging future research and development studies on biopolymer-based flocculants. The development of highly efficient, economical, safe, and multifunctional biopolymer-based flocculants will contribute to the emergence of sustainable and environmentally friendly alternatives that can replace their inorganic counterparts.

### 3.4. Structural Features and Morphology of Flocs

**Figure 10:**
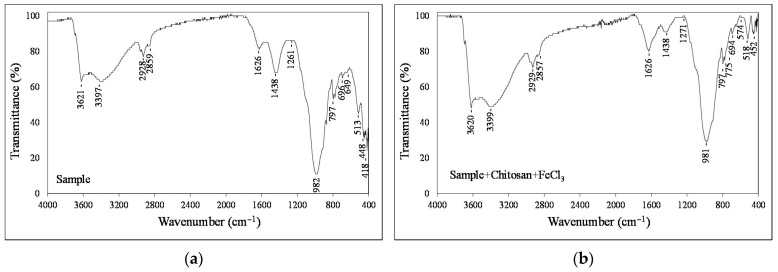
Infrared spectra of sample (**a**) and sample + chitosan + FeCl_3_ (**b**).

The FTIR spectra in Figure 10 show the functional groups of the sample and the functional group changes that occur after interaction of the sample with chitosan and FeCl_3_. The broad band observed at ~3621–3397 cm^−1^ is attributed to O–H stretching vibrations, commonly associated with hydroxyl groups on sample surfaces. After treatment with chitosan and FeCl_3_, transmittance decreased, indicating stronger hydrogen bonding and possible coordination interactions involving hydroxyl and amino groups. The absorption bands around 2929 and 2857 cm^−1^ correspond to asymmetric C–H bonds in aliphatic chains [15], while the peak at approximately 1626 cm^−1^ can be assigned to C=O stretching for the sample or N–H bending vibrations related to amide groups of chitosan [49]. The variation in the intensity of this band after interaction with Fe^3+^ suggests the participation of amino groups in coordination with metal ions. Furthermore, the strong band observed near 982 cm^−1^ is attributed to C–O stretching vibrations and possible Si–O contributions from mineral components in coal, consistent with previously reported methoxy or C–O related bands in polysaccharide-based systems [50]. The decrease in intensity of this band in the mixture indicates that the polymer coats the sample surfaces on the coal. The band observed at approximately 1438 cm^−1^ is attributed to the bending vibration of aliphatic CH_2_ groups. The variation in its intensity after treatment indicates the adsorption of polymer chains onto the sample surface and possible changes in the organic structure during the flocculation process. Overall, the observed changes in peak position and intensity confirm that chitosan adsorption and Fe^3+^ coordination occurred on the sample surface through hydroxyl and amino groups, suggesting that hydrogen bonding, surface complexation, and polymer bridging collectively contribute to the dewatering behavior of the sample.

**Figure 11:**
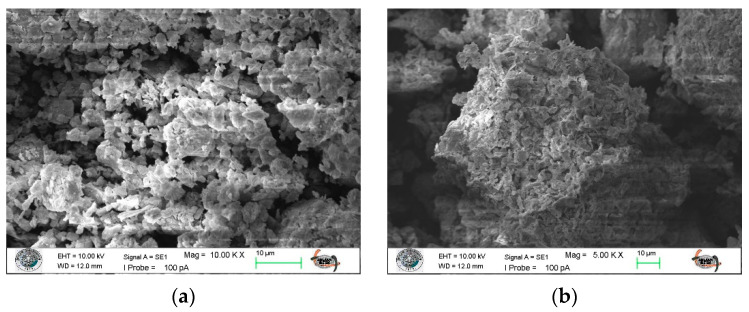
SEM micrographs showing the morphological changes in the sample (**a**) and the flocs formed after the addition of the chitosan-FeCl_3_ system (**b**).

The SEM micrographs provide direct evidence of the morphological changes occurring during the flocculation process. As depicted in Figure 11, these micrographs illustrate the morphology of the sample and its interaction with chitosan and FeCl_3_. In Figure 11a, the sample exhibits dispersed fine particles with irregular morphology and rough surfaces, indicative of a highly heterogeneous structure characteristic of mineral tailings enriched with clay and silicate minerals. The particles are observed to be loosely distributed without visible aggregation. Conversely, Figure 11b demonstrates that following the addition of chitosan and FeCl_3_, significantly larger and denser flocs are formed. The SEM images clearly show compact floc structures in which fine particles are strongly interconnected. This suggests that Fe^3+^ ions initially destabilize the negatively charged mineral surfaces through charge neutralization, thereby diminishing electrostatic repulsion between particles. Subsequently, chitosan chains facilitate bridging among the destabilized particles, resulting in larger and more stable flocs. The synergistic interaction between charge neutralization and polymer bridging leads to the formation of compact flocs, which are more favorable for rapid settling and efficient water recovery.

## 4. Conclusions

In this study, the flocculation behavior of coal preparation plant tailings was evaluated using the combination of an FeCl_3_–chitosan reagent system within statistically designed experimental conditions. Zeta potential analyses demonstrated that the sample exhibited a negative surface charge across the investigated pH range. Furthermore, chitosan effectively destabilized the suspension via charge neutralization and polymer bridging mechanisms. The role of FeCl_3_ was primarily through charge neutralization and the hydrolysis-induced formation of iron species, supporting floc aggregation under suitable pH and hydrodynamic conditions.

The results obtained from the experiments were modeled using RSM, and a strong correlation was observed between experimental data and the predicted values, evidenced by high R^2^ values. pH, pulp density, and chitosan dosage were identified as the most influential variables affecting turbidity and water recovery. The presence of statistically significant interaction terms highlights the importance of considering not only the individual effects of these parameters but also their combined chemical and operational interactions.

Optimization studies demonstrated that low pulp density, near-neutral pH, moderate-to-high chitosan dosage, and controlled stirring conditions provide superior clarification and dewatering performance. Under optimized conditions, turbidity was reduced to 9.86 NTU and water recovery reached 76.92%, confirming the effectiveness of the integrated reagent system. The FTIR and SEM analyses confirmed that chitosan adsorption and Fe^3+^ coordination occur on the sample surface through hydroxyl and amino functional groups, promoting particle destabilization and aggregation. The combined charge neutralization by Fe^3+^ ions and polymer bridging by chitosan resulted in the formation of dense and compact flocs, significantly enhancing settling behavior and water recovery. Overall, the findings provide a statistically validated framework for improving the dewatering of coal preparation plant tailings, supporting more sustainable water management in coal preparation plants.

## Figures and Tables

**Table 1:** Chemical composition of the sample.

Composition	Al_2_O_3_	CaO	Cr_2_O_3_	Fe_2_O_3_	K_2_O	MgO	Na_2_O	P_2_O_5_	SO_3_	SiO_2_	TiO_2_	LOI *
Content, %	13.78	5.48	0.05	7.73	2.62	3.21	1.20	0.09	1.04	37.78	0.75	26.12

* LOI refers to the loss of ignition.

**Table 2:** Proximate analyses of the sample.

Proximate Analysis (wt.%, Dry Basis)				
	Total Moisture	Ash	Volatile Matter	Fixed Carbon
Sample	3.32	73.65	22.02	1.01

**Table 3:** Operating conditions for flocculation experiments.

Coded Variables	Factors	Levels
A	Chitosan dosage, g/t	0, 100 and 200
B	FeCl_3_ dosage, g/t	0, 250 and 500
C	pH	5, 8 and 11
D	Stirring speed, rpm	250, 500 and 750
E	Pulp density, %	5, 10 and 15

**Table 4:** Experimental factors and responses for the flocculation experiments.

Run no	Independent Variable In Coded Form					Independent Variable in Actual Form					Responses	
	A	B	C	D	E	A	B	C	D	E	Turbidity (NTU)	Water Rec. (%)
1	0	0	0	−1	0	100	250	8	250	10	306.5	46.07
2	−1	−1	1	1	1	0	0	11	750	15	6015	1.72
3	0	0	−1	0	0	100	250	5	500	10	31.6	41.88
4	0	0	0	0	0	100	250	8	500	10	393.5	39.37
5	−1	1	1	1	−1	0	500	11	750	5	2012.5	2.86
6	1	0	0	0	0	200	250	8	500	10	47.05	36.85
7	−1	1	−1	−1	−1	0	500	5	250	5	1360	67.10
8	−1	1	−1	1	1	0	500	5	750	15	1500	8.58
9	0	0	0	0	0	100	250	8	500	10	406.5	49.42
10	1	−1	1	−1	1	200	0	11	250	15	6370	2.57
11	1	−1	1	1	−1	200	0	11	750	5	555	61.38
12	0	0	0	1	0	100	250	8	750	10	306	41.88
13	−1	1	1	−1	1	0	500	11	250	15	6485	2.14
14	0	0	0	0	0	100	250	8	500	10	206	46.90
15	0	0	0	0	0	100	250	8	500	10	357.5	44.39
16	1	1	−1	−1	1	200	500	5	250	15	56.25	38.60
17	1	1	1	−1	−1	200	500	11	250	5	569.5	66.29
18	−1	−1	−1	1	−1	0	0	5	750	5	570.5	57.28
19	−1	−1	1	−1	−1	0	0	11	250	5	2600	2.46
20	1	−1	−1	1	1	200	0	5	750	15	100.7	12.01
21	0	0	0	0	−1	100	250	8	500	5	74.75	81.43
22	1	−1	−1	−1	−1	200	0	5	250	5	21.65	69.56
23	0	0	0	0	0	100	250	8	500	10	391.5	45.23
24	0	1	0	0	0	100	500	8	500	10	210.5	45.23
25	0	0	0	0	1	100	250	8	500	15	596	14.15
26	0	0	1	0	0	100	250	11	500	10	5125	0.84
27	0	−1	0	0	0	100	0	8	500	10	522.5	46.90
28	−1	−1	−1	−1	1	0	0	5	250	15	745	10.29
29	1	1	1	1	1	200	500	11	750	15	5980	2.14
30	−1	0	0	0	0	0	250	8	500	10	1749	4.19
31	1	1	−1	1	−1	200	500	5	750	5	15.25	71.20
32	0	0	0	0	0	100	250	8	500	10	318	44.39

**Table 5:** ANOVA for the response surface quadratic model for turbidity and water recovery.

Response		Turbidity				Water Recovery			
Source of Variation	Df ^1^	Mean Square	F Value	*p* Value	Remarks	Mean Square	F Value	*p* Value	Remarks
A	1	4,827,351	14.56	0.003	Significant	2311.26	33.4	0.000	Significant
B	1	26,346	0.08	0.783		88.77	1.28	0.281	
C	1	54,465,676	164.3	0.000	Significant	3044.6	43.99	0.000	Significant
D	1	118,253	0.36	0.562		117.73	1.7	0.219	
E	1	22,375,385	67.49	0.000	Significant	8335.57	120.4	0.000	Significant
A^2^	1	297,641	0.90	0.364		776.91	11.23	0.006	Significant
B^2^	1	83,078	0.25	0.627		148.84	2.15	0.171	
C^2^	1	10,121,263	30.53	0.000	Significant	705.40	10.19	0.009	Significant
D^2^	1	146,494	0.44	0.52		79.48	1.15	0.307	
E^2^	1	113,608	0.34	0.57		222.09	3.21	0.101	
AB	1	214,683	0.65	0.438		35.29	0.51	0.49	
AC	1	7381	0.02	0.884		352.49	5.09	0.045	Significant
AD	1	32,901	0.10	0.759		21.94	0.32	0.585	
AE	1	617,621	1.86	0.200		703.81	10.17	0.009	Significant
BC	1	246,672	0.74	0.407		60.1	0.87	0.371	
BD	1	779,885	2.35	0.153		1170.55	16.91	0.002	Significant
BE	1	21,058	0.06	0.806		4.1	0.06	0.812	
CD	1	134,239	0.40	0.538		60.56	0.88	0.37	
CE	1	21,805,269	65.77	0.000	Significant	317.44	4.59	0.055	
DE	1	111,782	0.34	0.573		16.97	0.25	0.63	
Error	11	331,544				69.21			
Total	31	135,827,668				19,782.9			

^1^ Df = degrees of freedom.

**Table 6:** Favorable levels of the various factors for flocculation.

Factors	Turbidity (NTU)	Water Recovery (%)
A	0.798	0.515
B	−1	1
C	−0.152	−0.556
D	1	−1
E	−1	−1
Response	9.86	76.92

## Data Availability

The original contributions presented in this study are included in the article. Further inquiries can be directed to the corresponding author.

## Abbreviations

The following abbreviations are used in this manuscript:

FeCl_3_	Ferric chloride
RSM	Response Surface Methodology
R^2^	Coefficient of determination
PAC	Polyaluminum chloride
CMC	Carboxymethyl cellulose
–NH_2_	Amino
–OH	Hydroxyl
DOE	Experimental design
NaOH	Sodium hydroxide
HCl	Hydrochloric acid
ζ-potential	Zeta potential
NaCl	Sodium chloride
NTU	Nephelometric turbidity unit
ANOVA	Analysis of variance
